# Hepatotoxicity associated with sulfasalazine in inflammatory arthritis: A case series from a local surveillance of serious adverse events

**DOI:** 10.1186/1471-2474-9-48

**Published:** 2008-04-11

**Authors:** Paresh Jobanputra, Roshan Amarasena, Fiona Maggs, Dawn Homer, Simon Bowman, Elizabeth Rankin, Andrew Filer, Karim Raza, Ronald Jubb

**Affiliations:** 1 Department of Rheumatology, Selly Oak Hospital, University Hospital Birmingham NHS Foundation Trust, Raddlebarn Road, Birmingham, B29 6JD, UK; 2 Division of Immunity and Infection, MRC Centre for Immune Regulation, University of Birmingham, Birmingham, B15 2TT, UK

## Abstract

**Background:**

Spontaneous reporting systems for adverse drug reactions (ADRs) are handicapped by under-reporting and limited detail on individual cases. We report an investigation from a local surveillance for serious adverse drug reactions associated with disease modifying anti-rheumatic drugs that was triggered by the occurrence of liver failure in two of our patients.

**Methods:**

Serious ADR reports have been solicited from local clinicians by regular postcards over the past seven years. Patients', who had hepatotoxicity on sulfasalazine and met a definition of a serious ADR, were identified. Two clinicians reviewed structured case reports and assessed causality by consensus and by using a causality assessment instrument. The likely frequency of hepatotoxicity with sulfasalazine was estimated by making a series of conservative assumptions.

**Results:**

Ten cases were identified: eight occurred during surveillance. Eight patients were hospitalised, two in hepatic failure – one died after a liver transplant. All but one event occurred within 6 weeks of treatment. Seven patients had a skin rash, three eosinophilia and one interstitial nephritis. Five patients were of Black British of African or Caribbean descent. Liver enzymes showed a hepatocellular pattern in four cases and a mixed pattern in six. Drug-related hepatotoxicity was judged probable or highly probable in 8 patients. The likely frequency of serious hepatotoxicity with sulfasalazine was estimated at 0.4% of treated patients.

**Conclusion:**

Serious hepatotoxicity associated with sulfasalazine appears to be under-appreciated and intensive monitoring and vigilance in the first 6 weeks of treatment is especially important.

## Background

Spontaneous reporting systems for adverse drug reactions (ADRs), such as the yellow card system run by the Medicines and Healthcare products Regulatory Agency (MHRA) in the UK and the US Medwatch system, are handicapped by under-reporting and provide limited detail on individual cases. In an effort to learn more about ADRs associated with disease modifying anti-rheumatic drugs (DMARDs) we set up a local reporting system seven years ago[1] and reported preliminary findings in 2002 [2].

Case reports of serious hepatotoxicity associated with sulfasalazine are common yet only 161 ADRs attributed to the liver and biliary system have been reported to the Medicines and Healthcare products Regulatory Agency (MHRA) including nine fatalities [3]. In the US six patients developed drug related liver failure associated with sulfonamides in 2005 [4]. Drug 'hypersensitivity' reactions which include a morbilliform rash and hepatitis have, most recently, been called the DRESS (Drug Rash with Eosinophilia and Systemic Symptoms) syndrome [5,6] a term that places emphasis on eosinophilia. However serious toxicity without eosinophilia but including hepatitis is well recognised and older terms for this reaction, which occurs with other drugs such as carbamazepine and dapsone, have been called the 'three week syndrome' or the 'sulphone syndrome' [7,8].

Case reports are an important source of data which can guide management of new cases and trigger pharmaco-epidemiological investigations that may determine the probability of drug related toxicity. Drug related hepatotoxicity is an important cause of morbidity and mortality and indeed is the most common reason for withdrawing new drugs [9]. In this investigation, which was triggered by the occurrence of liver failure in two of our patients taking sulfasalazine, we describe a series of patients who developed serious hepatotoxicity whilst taking sulfasalazine.

## Methods

We have solicited reports of serious ADRs related to DMARDs from health care professionals treating patients with rheumatic disease in our institution by sending a postal or email postcard every 2 to 4 weeks over a period of 7 years. Details were requested for patients receiving DMARDs for RA or seronegative arthritidies. At inception we canvassed reports from colleagues in other local hospitals [2] but currently postcards and emails are targeted primarily at staff in our unit. We began in October 1999 when the first event was reported. A widely used definition of serious ADRs [10] was given in our early postcards but latterly any event regarded by a clinician as serious has been logged on our database. Clinicians are able to report serious events spontaneously or when prompted. This investigation was instigated when we became aware that two of our patients had developed liver failure whilst taking sulfasalazine.

Patients taking sulfasalazine and who developed a serious ADR which included hepatotoxicity were identified from our database. Identified cases were assessed for whether they met the definition of a severe or a life-threatening event according to the common toxicity criteria agreed by OMERACT (Outcome Measures in Rheumatology Clinical Trials) [11]. Thus, patients had to have major functional impairment of any duration associated with a rise in transaminases of at least 3 times the upper limit of normal, or a similar rise in alkaline phosphatase, or both. Concomitant jaundice was not necessary. One senior clinician made these judgments (PJ). For those meeting these criteria a detailed case report was prepared. The pattern of hepatatoxicity according to serum liver enzyme changes was classified as hepatocellular (transaminases above 2× normal alone or ratio of transaminases to alkaline phosphatase ≥ 5), cholestatic (increase in alkaline phosphatase above 2× normal or when ratio of enzymes is ≤ 2) or mixed according to agreed international criteria [12]. Case reports were reviewed by two senior clinicians who agreed, by consensus, whether the likelihood of drug-related hepatotoxicity was: highly probable; probable; possible; unlikely; or excluded. Two clinicians also used a detailed clinical index used for 'Causality Assessment of a Drug in a Case of Acute Liver Injury' [12,13]. This index was used in an effort to improve the probability that true drug related ADRs were identified [14]. The index includes domains for temporal relationship between drug use and adverse event, course of illness, consideration of other risk factors and search for non-drug causes, concomitant drug use and effects on re-challenge, if done.

We estimated the frequency of serious hepatotoxicity associated with sulfasalazine by making a series of conservative estimates and by using published data on the incidence of rheumatic diseases and the likely use of sulfasalazine.

## Results

### Case Series

In November 2006, when our analysis was done, our database had 203 reports of serious ADRs: 38 relating to sulfasalazine and 30 of these from one institution (University Hospital Birmingham). Nine other reported events were removed from the database because there was no suggestion of drug related toxicity. One patient who had developed upper gastrointestinal symptoms on sulfasalazine with abnormalities of liver function tests necessitating drug cessation was excluded as alanine transferase or alkaline phosphatase levels were not sufficiently raised (levels were below twice the upper limit of normal). Ten patients met our inclusion criteria for a serious adverse event: 8 reported during the surveillance period, 1 from some years previously and 1 reported by colleagues from a neighbouring hospital. By contrast, there were 63 reports of ADRs associated with methotrexate – 2 of these indicated liver disease, in one instance associated with alcohol excess.

The characteristics of the 10 patients with hepatitis whilst on sulfasalazine and their outcomes are shown in the Tables 1 &2. Five patients had a clinical diagnosis of RA, one possible RA, and the remaining 4 had sero-negative arthritidies. Six of the 10 patients had disease duration of less than a year. All patients had normal liver function tests before starting sulfasalazine including alkaline phosphatase, albumin, bilirubin and transaminases. None of the patients started any other new medications at the time of starting sulfasalazine. However all patients were using other drugs, in some cases potentially hepatotoxic medication such as diclofenac, when starting sulfasalazine (see Table 1). Eight of the 10 patients were hospitalised: three were admitted to the regional liver unit with jaundiced and two of these were in hepatic failure. Both the latter patients had a liver transplant but one died a few weeks after transplantion. Other causes of hepatitis were considered and investigated but exhaustive investigations were not done in all cases.

**Table 1:** Patient characteristics

Patient No./Age/Sex/Race*	Diagnosis* & Duration	**Time on drug^†^**	**Concomitant medication^‡^**	**Alcohol units/wk**
1/46/M/WB	PsA 20 years	2 days (On re-challenge)	Piroxicam, temazepam, paracetamol and dextropropoxyphene.	8 units
2/35/F/WB	RA 3 months	14 days	Diclofenac, paracetamol and dextropropoxyphene.	None
3/35/M/BB	ReA ~3 months	31 days	Diclofenac, flucloxacillin, paracetamol with dihydrocodeine	8 units
4/50/M/WB	PsA 28 months	>270 days	Felodipine, lansoprazole.	>30 units
5/33/F/BB	RA 23 months	~14 days	Celecoxib	None
6/30/F/WB	RA <3 months	5 days	Diclofenac. Methylprednisolone 120 mg intra-muscularly before starting sufasalazine.	None
7/64/F/BB	RA 10 years	~12 days	Chlorpheniramine, lisinopril, aspirin, cetirizine. Inhaled steroids & β-agonists	None
8/37/F/BB	RA 7 months	~42 days	Rofecoxib, prednisolone, paracetamol with dihydrocodeine, aspirin, folic acid.	None
9/45/F/WB	?RA 6 months	~17 days	Sertraline	None
10/48/F/BB	RA 3 months	10 days	Lansoprazole, tramadol, stemetil, thyroxine, rofecoxib, co-proxamol	None

* WB – White British, BB – Black British of African or Carribean descent. PsA: Psoriatic arthritis. RA: rheumatoid arthritis. ReA: reactive arthritis.

† For episode related to adverse event. ‡ At the time of adverse event.

**Table 2:** Clinical Features & Outcomes

**Pat. No**.	Clinical features# including liver enzymes**	**Probability^¶ ^& causality index score^§^**	**Outcome**
1	Systemically unwell, fever, rash. No eosinophilia. RF & ANA -ve. Transaminases >7× & alk. phos. >2× ULN. Bilirubin <2× ULN. Hepatocellular pattern. Hepatitis B & C status unknown.	Highly probable 4	Recovered
2	Nausea, dizziness, pruritis, rash, headache. No eosinophilia. RF -ve. ANA 1:400. Transaminases >2× & alk. phos. >8× ULN. Bilirubin <2× ULN. Mixed pattern. Hepatitis B & C status unknown.	Highly probable 2	Recovered
3	Lymphadenopathy, rash, fever, headaches, interstitial nephritis. Eosinophilia. RF & ANA -ve. Transaminases >4× & alk. phos. >2× ULN. Bilirubin <2× ULN. Mixed pattern. Hepatitis B & C status unknown.	Highly probable 6	Recovered. Given steroids.
4	Jaundice, systemically unwell. No eosinophilia. ANA-ve. RF not done. Transaminases >4× & alk. phos. >8× ULN. Bilirubin >10× ULN. Mixed pattern. Hepatitis B & C negative.	Possible 4	Recovered
5	Hepatic failure, rash, fever, diarrhoea. Lymphocytosis. No eosinophilia. ANA 1:40. RF +ve. Transaminases >50× & alk. phos. >1.5× ULN. Bilirubin >10× ULN. Hepatocellular pattern. Hepatitis B, C, & CMV negative.	Highly probable 8	Given steroids. Died after liver transplant
6	Hepatic failure preceded by nausea, vomiting, abdominal pain, diarrhoea. Eosinophilia. RF & ANA-ve. Transaminases >250× & alk. phos. >2× ULN. Bilirubin >6× ULN. Hepatocellular pattern. Hepatitis B, C & CMV negative.	Highly probable 9	Recovered after liver transplant
7	Abdominal pain, anorexia, nausea, rash, hypotension. Monocytosis. No eosinophilia. RF & ANA -ve. Transaminases >4× & alk. phos. >2.5× ULN. Bilirubin <2× ULN. Mixed pattern. Hepatitis B, C, & CMV negative.	Probable 7	Recovered
8	Nausea, vomiting, fever, rash, pruritus, sweating. Monocytosis & Eosinophilia. RF +ve. ANA 1:1600 post sulfasalazine. No pre-treatment value ANA. Transaminases >28× & alk. phos. >2.5× ULN. Bilirubin >2× ULN. Hepatocellular pattern. Hepatitis B, C & CMV negative.	Highly probable 7	Given steroids. Recovered
9	Lethargy, rash, dry cough, fever. Monocytosis. No eosinophilia. RF & ANA -ve. Transaminases >10× & alk. phos. >2× ULN. Bilirubin <2× ULN. Mixed pattern. Hepatitis B, C & CMV negative.	Highly probable 4	Recovered
10	Abdominal pain, nausea, vomiting, dizziness, palpitations, worsening joint pain. No eosinophilia. ANA 1:100. RF+ve. Transaminases >4× & alk. phos. >5× ULN. Bilirubin <2× ULN. Mixed pattern. Hepatitis B & C status unknown.	Possible 2	Given steroids. Recovered

# Eosinophilia refers to any value above normal. Some patients had a rise in eosinophil count above baseline but levels did not rise above the normal range. ** ULN = upper limit of normal. Pattern of toxicity classified as hepatocellular or mixed/cholestatic using published criteria [12].

¶Probability was determined by consensus and the clinical judgement of two senior clinicians according to a 5 point scale: highly probable, probable, possible, unlikely or excluded. & § Causality index scores were determined according to the methods described by Danan and Benichou (reference 12). A score of between -9 and +15 is possible on this scale: scores of <0 are considered to exclude drug toxicity; of 1–2 as 'unlikely'; 3–5 as 'possible'; 6–8 as 'probable' and over 8 'highly probable'. ANA: Anti-nuclear antibody. RF: rheumatoid factor. CMV:

All but three patients had liver and gallbladder ultrasound scans and/or an abdominal CT. Both patients with liver failure showed normal hepatic architecture on imaging. The third patient admitted to the liver unit showed a fatty liver. Gallstones were not seen. Serological tests for hepatitis B and C were done acutely in five of the ten patients and in a sixth a year later: tests were negative in all cases, details are shown in Table 2. Five patients had tests for cytomegalovirus, none tested positive for IgM. Patient 3, who had interstitial nephritis, was tested for human herpesvirus 6 and was negative.

Liver tissue from patient 5, who died, showed expanded portal tracts with a dense inflammatory infiltrate of mononuclear cells, mixed lymphocytes, plasma cells and some blast forms. Bile ducts showed similar inflammation and the liver parenchyma showed spotty infiltration with inflammatory cells and bridging necrosis. Hepatic veins showed subendothelial inflammation. An excess of eosinophils was not reported. Liver histology from patient 6, who also developed liver failure, showed massive hepatocyte necrosis with a mixed inflammatory cell infiltrate and little surviving parenchyma. Moderate numbers of eosinophils were noted. Variable portal tract inflammation was also seen. Other causes of liver failure such as Wilson's disease and alpha-1-antitrypsin deficiency were excluded in these two patients.

All but one event occurred within 6 weeks of starting treatment. Most patients felt generally unwell, 7 had a skin rash and one patient, with interstitial nephritis on biopsy, developed renal impairment (maximum creatinine 250 μmol/litre). Five of the 10 patients were Black British of African or Caribbean descent: racial type was assigned from personal knowledge of patients, hospital demographic data and by applying categories given in the 2001 census of England & Wales [15]. Liver enzymes showed a mixed pattern in 6 cases and a hepatocellular pattern in four. Likelihood of drug-related hepatotoxicity was judged highly probable in 7; probable in 1; and possible in 2 cases by two senior clinicians. Scores obtained by applying a published index for evaluating causality of drug related liver toxicity were poorly correlated with the clinical judgement of clinicians (see Table 2).

### Estimating the frequency of sulfasalazine related hepatotoxicity

We estimated the incidence of hepatotoxicity by making a series of assumptions which tend to underestimate the frequency of hepatotoxicity associated with sulfasalazine. The incidence of RA may be as high as 54 per 100000 female population and 25 per 100000 males [16]. The West Midlands, in England, has as many men as women and the city of Birmingham a population of around 977,000 [17]. One hundred and fifty eight new patients with RA per annum would be expected in the catchment area of University Hospital Birmingham (population served 400,000). We assumed that the other inflammatory arthritides combined contribute a further 42 patients per annum, bearing in mind that the incidence rate for psoriatic arthritis is between 3 to 8 per 100000 adults [18]. Our surveillance covers 7 years so potentially 1400 new patients may have developed an inflammatory arthritis. Assuming that all were referred to hospital, all given DMARDs, but that sulfasalazine was used in only 50%, based on a survey of UK practice [19], over 7 years 700 patients may have started sulfasalazine. The number of patients with prevalent inflammatory arthritis who also started sulfasalazine during this period cannot be estimated readily. However it is plausible that an equal number of patients with prevalent disease rather than newly diagnosed patients also started sulfasalazine i.e. 700 (the prevalence of RA for a population of 400,000 is estimated at 3200 [20]). Eight patients from the population served by University Hospital Birmingham developed serious hepatotoxicity during surveillance: 6 within one month of therapy. We estimate, therefore, that 0.4% (6/1400) of patients treated with sulfasalazine in our population developed serious hepatotoxicity: 4 were of a Black British background whereas around 12% of our catchment has this ethnic background [21] indicating that risk of hepatotoxicity is significantly higher in this ethnic group.

## Discussion

We estimated that the frequency of hepatitis with sulfasalazine was approximately 0.4% in our population. Two of our patients developed liver failure – an estimated rate of about 1.4 in 1000 people treated. It is possible that a higher than expected frequency of events occurred by chance during our surveillance and that the actual rate is lower: our estimates are in excess of those suggested by reviews of drug induced hepatotoxicity [22]; rates reported in observational studies; and data reported to the Committee for Safety of Medicines in the UK. For example, Ransford and Langman reported that 8.6 hepatitis reactions occurred per million prescriptions in RA patients compared with 3.1 per million prescriptions for people with inflammatory bowel disease [23]. Amos and colleagues, in another observational study, showed that only 2 of 774 patients stopped sulfasalazine because of abnormal liver enzymes [24]. However 26% of patients ceased therapy in their study within 1 year because of toxicity and a majority because of nausea, abdominal pain and vomiting: symptoms that were commonly encountered in our series with hepatitis and which often led to uncertainties about diagnosis. Despite reservations about our estimates, under reporting of serious adverse events, even for drugs under special surveillance, is well known and only half of all cases identified by prescription event monitoring are notified spontaneously [25]. Indeed a majority of the events related to sulfasalazine reported in this study (30 of 38) were from one institution indicating under-reporting from other centres involved at the beginning of our surveillance.

Most ingested sulfasalazine reaches the large bowel unchanged where it is split by colonic bacteria into sulphapyridine and 5-aminosalicylic acid (mesalazine). The latter remains in the large bowel but sulphapyridine is largely absorbed and eliminated in urine, principally after acetylation. Acetylation is determined by polymorphisms of the N acetyl transferase type II (NAT2) gene of which over 30 are known [26]. Between 40 and 70% of European and African populations, and less than a third of Far Eastern populations, are slow acetylators. Toxicity due to sulfasalazine including hepatitis appears, in general, to be more common in slow acetylators [27-29]. Thus an excess of Black British individuals with an African or Caribbean background in our series cannot readily be explained by current data for NAT2 genotype and phenotype and other explanations need to be considered. We do not know whether the reported excess of hepatitis and leucopenia attributed to sulfasalazine in RA patients compared with that in people with inflammatory bowel disease [23] is due to disease, patient characteristics, or reporting bias because of differences in monitoring practices between rheumatologists and gastroenterologists.

Drug induced hepatotoxicity may occur as a direct result of a drug or it's metabolites on hepatocytes or via immune activation. Reactions to sulfasalazine are believed to arise because of an idiosyncratic delayed-type hypersensitivity reaction that may affect internal organs variably. Fatalities may occur in 10% or more cases and are commonly due to liver involvement [30]. It is suggested that this is due to eosinophil infiltration of the liver [31]; eosinophilia on liver biopsy was noted in one of our patients with liver failure. However it is also reported that there are no specific histological features associated with drug induced hepatitis [32]. Treatment of drug related hepatotoxicity is supportive in most cases [30]. Cessation of suspected drugs is vital and steroids are commonly used although there is no clear consensus that they are effective [31]. Four of our patients were given steroids and the patient with liver failure who survived had received an intra-muscular injection of methylprednisolone just prior to starting sulfasalazine.

Toxicity without typical features of hypersensitivity, such as a skin rash, is well recognised [33]. Adverse reactions to sulphonamides are more common in the presence of viral infections such as human immunodeficiency virus [34] and perhaps human herpes simplex-6 (HHV6) although the one patient we tested for HHV6 was negative [35]. Systemic reactions to sulfasalazine and other drugs which include eosinophilia have been dubbed the DRESS syndrome. Associated interstitial nephritis is less common and likely to be due to mesalazine [23]. In our series eosinophilia was not invariable and we concur with Peyriére and colleagues that this term is unnecessarily restrictive [36]. The term 'three week syndrome' is older and accurately describes the timing of serious toxicity: it is worth noting that both the British Society for Rheumatology guidelines [37] and the summary of product characteristics for salazopyrin [38] recommend that liver function tests should be monitored every 4 weeks initially. Thus a majority of our patients would not have been identified by national monitoring recommendations. Our practice is prescribe sulfasalazine at 500 mg per day in week 1 and increase weekly by 500 mg up to 2 g per day at week 4. We monitor blood every 2 weeks for the first 12 weeks of therapy [39]. This policy was in place at the time these events occurred and no deviations from our policy were identified. The occurrence of these events has led some local clinicians to argue for weekly monitoring during the first month of therapy especially in patients with a Black British background.

Determining causality, in cases of suspected drug induced hepatitis, can be difficult not least because of concomitant therapy including possible synergistic effects between potentially hepatotoxic drugs and alcohol use. One of our patients was known to consume alcohol to excess and a majority on other potentially hepatotoxic drugs such as diclofenac. Diagnostic work-up in our patients also varied greatly and, understandably, appeared to depend on the severity of liver disease. In some cases limited diagnostic testing appeared to reflect greater clinical certainty in the diagnosis of a drug induced reaction. However the absence of a comprehensive work up in all cases hampers retrospective judgments about causality. Various indices have been developed to assess causality which rely on a scoring system but there is no agreed gold standard and agreement between different scales is often poor [13]. We tested the causality assessment index described by Danan and Benichou [12] (see Table 2), regarded as essential in drug induced hepatotoxicity studies [32]. We also used a simple consensus method similar to the preferred judgments of hepatologists [40]. There was a poor relationship between our consensus method and the causality assessment index, in part because of dependence on a positive re-challenge to attain maximum scores with the causality index. Only one of our patients was re-challenged and developed an adverse reaction with 48 hours. Clinicians should beware of re-challenging patients with suspected hypersensitivity reactions because of reports that even a small dose on rechallenge can precipitate massive hepatic necrosis [32,41].

## Conclusion

We conclude that serious hepatotoxicity often associated with a skin rash and variably associated with eosinophilia occurs within 6 weeks of starting sulfasalazine therapy. Our experiences indicate that such toxicity is likely to be missed by current national recommendations for monitoring. Toxicity in our series was particularly common in people of a Black British background of African or Caribbean descent. We believe that this adverse drug reaction is underappreciated and may occur with a frequency as high as 0.4%. Although this risk frequency is regarded by convention as uncommon the potential severity of reactions seen may make some patients and practitioners wary of sulfasalazine.

## Competing interests

The author(s) declare that they have no competing interests.

## Authors' contributions

PJ and RA collated the case reports, extracted data, assigned causality scores and drafted the abstract. PJ conceived the project and drafted this manuscript. FM compiled the database, sought adverse events and did initial analyses. DH, SB. ER, AF, KR, RJ and PJ contributed cases and participated in study discussions. All authors read and approved the final manuscript.

## Pre-publication history

The pre-publication history for this paper can be accessed here:


